# Complete genome sequence of *Bacillus cereus* phage vB_BceH_LBC2, a lytic member of the genus *Bequatrovirus*

**DOI:** 10.1128/mra.00762-25

**Published:** 2025-10-17

**Authors:** Eo-Jin Kim, Jeong-A Lim

**Affiliations:** 1 Food Safety and Distribution Research Group, Korea Food Research Institute 71645, Wanju-gun, Republic of Korea; 2 Department of Food Science and Biotechnology, Chung-Ang University 65479 , Anseong-si, Republic of Korea; Queens College Department of Biology, Queens, New York, USA

**Keywords:** bacteriophage, genome sequencing, *Bacillus cereus*, *Bequatrovirus*

## Abstract

Phage vB_BceH_LBC2 is a lytic bacteriophage infecting *Bacillus cereus*, classified as a member of the genus *Bequatrovirus* (family *Herelleviridae*). Its genome is 157,419 base pairs, with 232 predicted genes and a GC content of 35.63 percent. No virulence, antibiotic resistance, or lysogeny-related genes were identified.

## ANNOUNCEMENT

*Bacillus cereus* is a gram-positive, spore-forming bacterium linked to food spoilage and gastrointestinal illness (1). Its ubiquity and resistance make it a frequent target for phage-based control (2). Lytic phages offer host-specific antibacterial potential (3). Here, we report the isolation, morphology, and genome sequence of lytic phage vB_BceH_LBC2, a member of the genus *Bequatrovirus*.

Phage vB_BceH_LBC2 was isolated from sewage samples from wastewater treatment plants in Iksan, South Korea (35.988558°N, 126.935605°E), using *B cereus* NCCP 14796, obtained from the National Culture Collection for Pathogens (NCCP), as the host strain. Filtered samples were mixed with 2× TSB containing 2 mM MgCl_2_ and CaCl_2_, incubated with the host at 37°C for 24 h, re-filtered, and spotted onto *B. cereus* lawns via double-layer agar (4). Plaques were picked and purified through three reinfection rounds. Phages were propagated in exponential-phase cultures, lysate dialyzed with SM buffer, and stored at 4°C (5).

**Fig 1:**
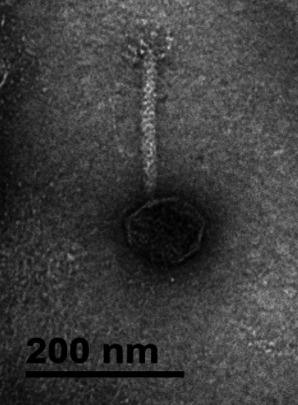
Transmission electron micrograph of *B. cereus* phage vB_BceH_LBC2. Phage particles were negatively stained with 2% (wt/vol) uranyl acetate (pH 4.0) and imaged using a Hitachi H-7650 transmission electron microscope (Hitachi, Tokyo, Japan) at Jeonbuk National University (Jeonju, Republic of Korea), operated at 80 kV accelerating voltage and ×100,000 magnification. Particle dimensions were analyzed using ImageJ v1.52 (NIH, Bethesda, MD, USA). Scale bar = 200 nm.

Phage morphology was confirmed by transmission electron microscopy (TEM) after negative staining with 2% (wt/vol) uranyl acetate (pH 4.0) (6). TEM showed LBC2 possesses an icosahedral capsid (length: 86.3 ± 1.2 nm; width: 90.9 ± 6.0 nm) and a long, flexible tail (205.7 ± 5.8 nm), typical of siphovirus-like morphology (Fig. 1).

Genomic DNA was extracted from a high-titer phage stock using the Phage DNA Isolation Kit (Norgen Biotek, Ontario, Canada) (7). Paired-end libraries (2 × 300 bp) were prepared using the TruSeq Nano DNA Library Prep Kit (Illumina) and sequenced on the Illumina MiSeq platform by Sanigen Co., Ltd. (South Korea). Adapter trimming and quality filtering were performed using Trimmomatic v0.39 (8), and read quality was assessed with FastQC v0.11.9. A total of 852,458 reads were generated, yielding approximately 242 Mb of sequence data, with an average genome coverage of ~1,537× . To assess the genome termini and packaging strategy, we analyzed the raw sequencing reads using PhageTerm (9). *De novo* genome assembly was performed using SPAdes v3.15.2 with default parameters (10). All other software was run with default parameters unless otherwise specified. Open reading frames (ORFs) were predicted using GeneMarkS (11), and annotated using RAST (12) and BLASTp searches against the NCBI non-redundant protein database (13). In addition, tRNA and tmRNA genes were searched using ARAGORN v1.2.41 (14).

The complete genome of phage LBC2 is 157,419 bp in length and has a GC content of 35.63%. The genome comprises 232 predicted protein-coding sequences, of which 99 were assigned putative functions. PhageTerm analysis suggested that the phage genome is circularly permuted with multiple preferred termini, consistent with a headful packaging mechanism. No tRNA genes or other non-coding RNA elements were identified in the genome. No virulence or lysogeny-associated genes, including integrases or repressors, were detected. Based on sequence similarity and current ICTV taxonomy (15), phage LBC2 is classified within the genus *Bequatrovirus*, subfamily *Bastillevirinae*, family *Herelleviridae*, and class *Caudoviricetes*. The closest known relative to phage LBC2 is *Bacillus* phage vB_BceH_LY2 (GenBank accession no. ON366411.1), with 97.68% nucleotide identity over 82% of the genome, supporting its classification within the genus *Bequatrovirus*.

## Data Availability

The complete genome sequence of phage LBC2 was deposited in GenBank under accession number PV449003. The associated BioProject, BioSample, and SRA accession numbers are PRJNA1244569, SAMN47853738, and SRR33046712, respectively.
